# Stroke-Induced Modulation of Myeloid-Derived Suppressor Cells (MDSCs) and IL-10-Producing Regulatory Monocytes

**DOI:** 10.3389/fneur.2020.577971

**Published:** 2020-11-25

**Authors:** Lennart Achmus, Johanna Ruhnau, Sascha Grothe, Bettina von Sarnowski, Barbara M. Bröker, Alexander Dressel, Juliane Schulze, Antje Vogelgesang

**Affiliations:** ^1^Department of Neurology, University Medicine Greifswald, Greifswald, Germany; ^2^Department of Diagnostic Radiology and Neuroradiology, University Medicine Greifswald, Greifswald, Germany; ^3^Department of Immunology, University Medicine Greifswald, Greifswald, Germany; ^4^Department of Neurology, Carl-Thiem Klinikum, Cottbus, Germany

**Keywords:** MDSC (myeloid-derived suppressor cell), IL10, ischemic stroke, experimental stroke, immune suppression, regulatory monocytes

## Abstract

**Background:** Stroke patients are at risk of acquiring secondary infections due to stroke-induced immune suppression (SIIS). Immunosuppressive cells comprise myeloid-derived suppressor cells (MDSCs) and immunosuppressive interleukin 10 (IL-10)-producing monocytes. MDSCs represent a small but heterogeneous population of monocytic, polymorphonuclear (or granulocytic), and early progenitor cells (“early” MDSC), which can expand extensively in pathophysiological conditions. MDSCs have been shown to exert strong immune-suppressive effects. The role of IL-10-producing immunosuppressive monocytes after stroke has not been investigated, but monocytes are impaired in oxidative burst and downregulate human leukocyte antigen—DR isotype (HLA-DR) on the cell surface.

**Objectives:** The objective of this work was to investigate the regulation and function of MDSCs as well as the immunosuppressive IL-10-producing monocytes in experimental and human stroke.

**Methods:** This longitudinal, monocentric, non-interventional prospective explorative study used multicolor flow cytometry to identify MDSC subpopulations and IL-10 expression in monocytes in the peripheral blood of 19 healthy controls and 27 patients on days 1, 3, and 5 post-stroke. Quantification of intracellular STAT3p and Arginase-1 by geometric mean fluorescence intensity was used to assess the functionality of MDSCs. In experimental stroke induced by electrocoagulation in middle-aged mice, monocytic (CD11b^+^Ly6G^−^Ly6C^high^) and polymorphonuclear (CD11b^+^Ly6G^+^Ly6C^low^) MDSCs in the spleen were analyzed by flow cytometry.

**Results:** Compared to the controls, stroke patients showed a relative increase in monocytic MDSCs (percentage of CD11b^+^ cells) in whole blood without evidence for an altered function. The other MDSC subgroups did not differ from the control. Also, in experimental stroke, monocytic, and in addition, polymorphonuclear MDSCs were increased. The numbers of IL-10-positive monocytes did not differ between the patients and controls. However, we provide a new insight into monocytic function post-stroke since we can report that a differential regulation of HLA-DR and PD-L1 was found depending on the IL-10 production of monocytes. IL-10-positive monocytes are more activated post-stroke, as indicated by their increased HLA-DR expression.

**Conclusions:** MDSC and IL-10^+^ monocytes can induce immunosuppression within days after stroke.

## Introduction

Ischemic stroke not only destroys brain tissues but also induces strong immune suppression. Stroke-induced immune suppression (SIIS) results in increased rates of secondary infections, increasing mortality and worsening patient outcomes (1, 2). While the last decade of research has revealed the impact of SIIS on the major immune cell subpopulations, regulatory subsets like interleukin 10 (IL-10)-producing monocytes and myeloid-derived suppressor cells (MDSCs) have not been investigated in detail.

MDSCs are a heterogeneous population consisting of early myeloid progenitors and precursors of granulocytes, macrophages, and dendritic cells (DCs). MDSCs regulate the innate and adaptive immune system and suppress T cell-mediated immune responses (3, 4). In the context of infection or tissue destruction, myelopoiesis is enhanced, which leads to the liberation of immature progenitor cells from the bone marrow. In stroke, these mechanisms can be enhanced by the direct impact of the nervous system through the innervation of the bone marrow (5). Murine MDSCs are characterized by the co-expressions of CD11b and Gr-1 and can be further distinguished by the expressions of Ly-6G and Ly-6C into granulocytic or monocytic MDSCs (6). In contrast, the phenotypic characterization of human MDSCs is extremely heterogeneous (4) in the literature. Nevertheless, they can be grouped into three major subpopulations by their surface marker expression: (i) monocytic MDSCs (Mo-MDSCs; CD11b^+^/CD14^+^/HLA-DR^dim^); (ii) granulocytic or polymorphonuclear MDSCs (PMN-MDSCs; CD11b^+^/CD14^−^/CD15^+^); and (iii) early MDSCs (e-MDSCs; Lin^−^/HLA-DR^−^/CD33^+^/CD11b^+^) (4) (Supplementary Figure 1). Mo-MDSCs can differentiate into macrophages or dendritic cells (3, 4, 7, 8) and are the most immunosuppressive subset of MDSCs (3, 4, 7, 8).

Increased amounts of splenic CD11b^+^Ly-6C^+^ Mo-MDSCs with upregulated arginase-I (Arg1) as a marker for an intact immunosuppressive capacity were reported in an experimental stroke model after 24 h (9). The same study detected an increase of e-MDSCs in a group of six stroke patients 24 h after ischemic stroke (9).

The transcription factor STAT3p and the substrate l-arginine can be used to characterize the functional status of MDSCs. STAT3p induces immunosuppressive MDSCs (10, 11) and is responsible for reactive oxygen species (ROS) production in PMN-MDSCs as well as their expansion in a pro-inflammatory environment (12). The non-essential amino acid l-arginine is the substrate of nitric oxide synthase and Arg1 that deplete l-arginine from the microenvironment, resulting in the prevention of T cell activation, inhibition of T cell proliferation, and induction of apoptosis (13, 14).

In stroke, monocytes in general are not reduced in number but are deficient for tumor necrosis factor-α production and human leukocyte antigen—DR isotype (HLA-DR) expression (15, 16), which is important for antigen presentation to the adaptive immune system. While the immunoregulatory properties of developed monocytes are well-known, the contribution of IL-10-producing regulatory monocytes to SIIS remains largely unknown, though their potential for the induction of an immune suppression has been demonstrated (17).

D'Aveni et al. (18) described a population of CD34^+^ monocytes endowed with potent immunosuppressive activity by nitric oxide (NO) production and subsequent induction/generation of T regulatory cells. These cells, characterized by CD34^+^Lin^−^HLA-DR^−^CD33^+^CD11b^+^CD14^+^, were induced by granulocyte colony-stimulating factor (G-CSF) and exerted a very strong immunosuppressive effect on T cell activation. Indeed, G-CSF can induce and mobilize several subsets of granulocytes and monocytes with immunoregulatory properties (19–21) and is released after stroke in higher amounts (22).

To functionally characterize IL-10-producing monocytes, we quantified the surface molecules PD-L1/2, HLA-DR, and CD86.

This study analyzed MDSC subsets and the functionally important STAT3p and Arg1 as well as regulatory IL-10^+^ monocytes. We found an increase of Mo-MDSCs in the experimental stroke model and in human stroke patients in comparison to healthy controls. We were unable to define regulatory monocytes based on the markers suggested by D'Aveni et al. (18) but characterized IL-10^+^ monocytes after stroke. We here describe for the first time that these cells exhibit differential expressions of HLA-DR and PD-L1 in comparison to IL-10^−^ monocytes in stroke patients.

## Methods

### Human Data

#### Patients and Control Samples

For this longitudinal, monocentric, non-interventional prospective explorative study, 27 ischemic stroke patients from the dedicated stroke unit of the University Medicine Greifswald were recruited from 2017 to 2018. Blood was sampled on days 1, 3, and 5 after admission to the stroke unit. The numbers differ for each experimental protocol performed in this study. Twenty-seven stroke patients were compared to 16 healthy age-matched controls for the extracellular MDSC protocol (Supplementary Table 1), while 10 stroke patients were compared to 10 healthy age-matched controls for the intracellular MDSC protocol (Supplementary Table 2). For the characterization of human monocytes, 12 stroke patients were compared to 10 healthy age-matched controls (Supplementary Table 3).

All patients' characteristics are provided in Table 1.

**Table 1:** Patient characteristics.

**Variable**	**Patient group (*N =* 27)**	**Control group (*N =* 19)**
Age [Years, Mean ± SD]	70.2 ± 13.4	67.2 ± 9.3
Sex [as % female]	40.7	31.6
**Co-morbidities**		
Hypertension [n (%)]	23 (85.19)	15 (78.95)
Diabetes mellitus [n (%)]	10 (37.04)	8 (42.11)
**Stroke characteristics**		
Etiology		
Large-artery atherosclerosis [n (%)]	3 (11.11)	NA^$^
Cardio embolism [n (%)]	9 (33.33)	NA^$^
Stroke of other determined etiology [n (%)]	5 (18.52)	NA^$^
Stroke of undetermined etiology [n (%)]	10 (37.04)	NA^$^
1. MRI Stroke Size^*^ [ml3, Median (IQR)]	2,88 (4.83)	NA^$^
Initial NIHSS [Median (IQR)]	11 (7)	NA^$^
NIHSS at discharge [Median (IQR)]	4 (7.5)	
Infarct side [n (%) left sided infarcts]	19 (70.37)	NA^$^
Treatment [n (%)]	19 (70.37)	NA^$^
Systemic Thrombolysis [n (%)]^&^	17 (89.47)	NA^$^
Mechanical Thrombectomy [n (%)]^&^	10 (52.63)	NA^$^
Combined Treatment [n (%)]^&^	8 (42.11)	NA^$^

& *The numbers of systemic thrombolysis and mechanical thrombectomies are the total number of patients receiving the treatments and include patients receiving a combination of both*.

$ *NA, Not applicable*.

* *Stroke size could be determined in 7 out of the 27 patients*.

Patients aged ≥18 years suffering from middle cerebral artery (MCA) infarct were eligible for this study within 24 h after the onset of symptoms if their National Institutes of Health Stroke Scale (NIHSS) score was ≥6 and if no signs of systemic infection were detected on admission [C-reactive protein (CRP) ≤ 50 mg/L and procalcitonin (PCT) ≤0.5 ng/ml]. Patients receiving immunosuppressive drugs or diagnosed with a malignancy or an autoimmune disease were not recruited. Additional exclusion criteria were severe cerebral medical conditions within the past 6 months, clinically significant anemia, immunosuppression, and no written informed consent by the patient himself or through a surrogate, where appropriate.

Patients were treated with the best medical care on a dedicated stroke unit. Recanalization with recombinant tissue plasminogen activator (r-tPA) and/or thrombectomy was carried out as clinically indicated.

Age-matched control individuals were recruited from ophthalmology clinics among patients in treatment for age-related macular degeneration or scheduled to receive cataract surgery. Controls were neurologically and immunologically healthy. The study was approved by the local ethics committee (No. BB 041/17).

#### Blood Sampling

Blood was obtained in different quantities using Li-heparin between 7:00 and 8:00 in the morning on days 1, 3, and 5 after admission to the stroke unit. CRP (measured by the Dimension Vista, Siemens Healthcare Diagnostics, Eschborn, Germany) was measured in the central laboratory facility of the University Medicine Greifswald.

#### Determination of Stroke Lesion Size

Diffusion-weighted magnetic resonance imaging (MRI) images were used to calculate infarct sizes (3.0 T). To determine the location and stroke size, the images were analyzed using OSIRIX 5.6. To calculate the infarct size, the regions of interest were defined manually, and the lesion volume was calculated semi-automatically.

Patients underwent MRI only as clinically indicated and not study-related. Consequently, lesion size is only available for a limited number of seven patients.

#### FACS of Human MDSCs

Heparinized whole blood samples for “fluorescence-activated cell sorting (FACS)” analysis of human MDSC subsets were obtained using BD-Vacutainer systems (Li-heparin, 17 IU/ml) on days 1, 3, and 5 after admission to the stroke unit. Peripheral blood mononuclear cells (PBMCs) were isolated within 30 min using 1,077 g/ml Biocoll separating solution (Biochrome GmbH, Berlin, Germany) at room temperature. All reagents were obtained from BioLegend Inc. (San Diego, CA, USA), unless otherwise indicated.

Extracellular staining of MDSC subsets was performed using monoclonal antibodies: anti-CD11b BV510 (clone M1/70, 1:20), anti-CD33 BV421 (clone WM53, 1:50), anti-CD14 PE/Cy7 (clone 63D3, 1:50), anti-CD15 AF700 (clone W6D3, 1:20), and anti-HLA-DR fluorescein isothiocyanate (FITC; clone L243, 1:50). To exclude mature leukocyte subsets, a lineage cocktail (APC, 1:5) containing monoclonal antibodies against anti-CD3, anti-CD14, anti-CD16, anti-CD19, anti-CD20, and anti-CD56 (clones UCHT1, HCD14, 3G8, HIB19, 2H7, and HCD56) was used. Before staining, unspecific binding of the antibodies was blocked using an Fc receptor (FcR) blocking reagent (1:10; Miltenyi Biotec GmbH, Bergisch Gladbach, Germany). For functional analysis, intracellular staining of MDSC subsets included an anti-arginase-1 PE (clone 14D2C43, 1:50) and phosphorylated transcription factor anti-STAT3 phospho PerCP/Cy5.5 (clone 13A3–1, 1:20). Fixation and permeabilization of cells were performed using True-Nuclear transcription buffer set according to the manufacturer's instructions. The cells were measured using a BD LSRII flow cytometer. Data analysis was performed using FlowJo10 (FlowJo LLC, Ashland, OR, USA). Gating of the MDSC subpopulations is shown in Supplementary Figure 2.

#### FACS of Human Monocytes

Heparinized whole blood samples for FACS analysis of human monocytes were obtained using BD-Vacutainer systems (Li-heparin, 17 IU/ml) on days 1 and 5 after admission to the stroke unit. PBMCs were isolated as described above. The cells were then counted using a Neubauer cell counting chamber and diluted to a final concentration of 2 × 10^6^/ml using X-Vivo15 serum-free medium (Lonza Group Ltd., Basel, Switzerland). About 2 × 10^6^ cells per well were placed in a 24-well chamber (Corning Costar, Sigma-Aldrich Corp., St. Louis, MO, USA) and incubated for a total of 20 h without or with 10 ng/ml lipopolysaccharide (LPS) to induce cytokine production (Sigma-Aldrich Corp.). All reagents were obtained from BioLegend Inc., unless otherwise indicated. Brefeldin A was added after 12 h of stimulation at a concentration of 1:1,000 to prevent cytokine excretion through the Golgi apparatus. Following stimulation, the cells were washed and prepared for FACS analysis.

To exclude dead cells from our analysis, Zombie Yellow Fixable Viability Dye was applied to the cells prior to extracellular staining. To prevent unspecific binding, FcR blocking reagent (1:10; Miltenyi Biotec GmbH) was added prior to extracellular staining. Extracellular staining was done by anti-CD11b BV510 (clone M1/70, 1:50), anti-CD34 BV421 (clone 561, 1:20), anti-CD14 APC/Cy7 (clone HCD14, 1:50), anti-CD86 BV650 (clone IT2.2, 1:20), anti-HLA-DR FITC (clone L243, 1:50), anti-CD33 PerCP/Cy5.5 (clone WM53, 1:50), anti-PD-L1 PE/Dazzle594 (clone 29E.2A3, 1:50), and anti-PD-L2 APC (clone MIH18, 1:20) antibodies. For intracellular staining, anti-IL-10 PE (clone JES3–9D7, 1:50) was added. Fixation and permeabilization of cells were achieved by using True-Nuclear transcription buffer set according to the manufacturer's instructions. FACS analysis was performed using a BD LSRII cytometer. Data analysis was performed using FlowJo10 (FlowJo LLC). Gating of monocytes, including IL-10 gating, is illustrated in Supplementary Figure 3.

### Mouse Data

#### Animals

All animal experiments were approved by the local government authorities [Landesamt für Landwirtschaft, Lebensmittelsicherheit und Fischerei (LALLF), Mecklenburg-Vorpommern, nr. 7221.3-1-056/15]. Group size was calculated prior to experiments. Mice were purchased from Janvier Labs (Le Genest-Saint-Isle, France). In our study, we used male C57Bl/6 mice to exclude variations due to estrous cycle influences. Two weeks prior to the start of the experiments, the animals were delivered from Janvier and housed in the small animal MRI facility and allowed to adapt. Mice were group-housed with an enriched environment (red transparent plastic boxes and paper rolls). All Animals were kept under a 12-h light/dark cycle (6:00 a.m., 6:00 p.m.) and had *ad libitum* access to standard chow and acidified tap water. Room temperature and humidity were monitored and kept at 22 ± 1°C and 50–60%, respectively.

#### Ischemia Model

Fourteen-month-old male C57Bl/6 mice underwent coagulation of the left distal middle cerebral artery using the electrocoagulation model. Stroke size for the day 1 cohort was 2.85 mm^2^ (mean, min–max = 1.60–7.13 mm^2^) and for day 3 was 5.05 mm^2^ (mean, min–max = 2.94–8.76 mm^2^). The electrocoagulation was always performed between 8:00 and 11:00 a.m. Mice underwent permanent coagulation of the distal middle cerebral artery. Anesthesia was induced with 2.5% isoflurane with a 1-L/min N_2_O/O_2_ mix (ratio, 0.7:0.3) and maintained at 2% isoflurane during surgery. Body temperature was measured with a rectal probe and maintained at ≥36.5°C using a feedback-controlled heating pad. After the skin was incised between the left eye and ear, the temporal muscle was incised and retracted. The lenticulostriatal branches were located underneath the skull. The skull was thinned using a dental drill. The skull and dura were carefully removed to avoid bleeding. The middle cerebral artery was carefully electrocoagulated above the lenticulostriatal branches using a cauterizer. The temporal muscle flap was relocated above the burr hole, the skin wound was closed with a simple interrupted suture, and lidocaine gel was applied topically for pain relief. The total surgery time did not exceed 15 min. The animals were kept in a warming chamber until the next morning and were provided with additional soft, mashed chow in a Petri dish on the floor of the chamber as well as free access to water. After surgery, the body temperature and body weight, as well as scoring, were assessed on a daily basis in the morning.

#### Mouse MRI

For 7-T animal MRI (ClinScan, Bruker Biospin, Ettlingen, Germany), the mice were anesthetized with 1–2% isoflurane and 1 L/min oxygen. During brain scans, respiration was monitored and the animals were kept warm using an external water bath. For brain scans at day 1 after middle cerebral artery occlusion (MCAO), a 3D T2-weighted imaging (mouse brain coil, TR = 2,000 ms, TE = 37 ms, FoV = 19 × 25 mm, 0.45 mm thickness) and additional diffusion-weighted imaging for visualization of the acute infarct were performed. For evaluation of the T2 lesion volume of the brain, MRI data were analyzed by two independent investigators with respect to the lesion location and size. Regions of interest were selected manually, and the volume was calculated semiautomatically using OsiriX software.

#### FACS of Mice

The spleens were removed from donor animals at the time of sacrifice and processed into single cell suspensions. For FACS staining, the cells were transferred into a FACS buffer and first incubated with Fc block (BD Biosciences). All reagents were obtained from BioLegend Inc., unless otherwise indicated.

For defining T cell subpopulations (compare Supplementary Figure 5), the following panels were used: anti-mouse CD4 FITC (clone RM4–5, 0.5 mg/ml; Biolegend), anti-CD25 PE-Cy7 (clone PC 61, 0.2 mg/ml; Biolegend), anti-mouse CD69 PE (clone H1.2F3, 0.2 mg/ml; Biolegend), anti-mouse CD11b APC (clone M1/70, 0.2 mg/ml; BD), anti-mouse FoxP3 (clone 3G3, 0.2 mg/ml; Miltenyi), anti-mouse CD11b FITC (clone N418, 0.5 mg/ml; Biolegend), anti-mouse CD4 PE (clone RM4–5, 0.2 mg/ml; Biolegend), anti-mouse MHC II (clone M5/114.15.2, 0.5 mg/ml; eBioscience), anti-mouse CD19 Alexa Fluor 647 (clone 6D5, 0.5 mg/ml; Biolegend), anti-mouse Ly6G (clone 1A8, 0.2 mg/ml; BD), anti-mouse B220 (clone RA3–6B2, 0.2 mg/ml; eBioscience), streptavidin PE-Cy7 (0.2 mg/ml; Biolegend), anti-mouse CD4 A700 (clone GK 1.5, 0.5 mg/ml; Biolegend), anti-mouse CD8 Biotin (clone 53–6.7, 0.5 mg/ml; Biolegend), anti-mouse CD3e V500 (clone 500A2, 0.2 mg/ml; BD), and anti-mouse CD11b Alexa Fluor 780 (clone M1/70, 0.2 mg/ml; eBioscience).

For the staining of polymorphonuclear and monocytic MDSCs (PMN-MDSC and Mo-MDSC), splenocytes were stained with the following antibody panel: anti-mouse F4/80 PE-Cy7 (clone BM8, 1:50; Biolegend), anti-mouse CD13 BV510 (clone R3–242, 1:50; BD), anti-mouse Ly6G V421 (clone 1A8, 1:50; Biolegend), anti-mouse Ly6C BV605 (clone HK 1.4, 1:50; BD), anti-mouse CD3 PerCP/Cy5.5 (clone 17A2, 1:50; Biolegend), anti-mouse CD62L FITC (clone MEL-14, 1:50), anti-mouse CD43 PE (clone S11, 1:50; Biolegend), and anti-mouse CD11b APC-Cy7 (clone M1/70, 1:50; Biolegend). Because of the *post-hoc* characteristic of this study, the FACS panel includes more markers than analyzed within this paper. We report the whole staining panel since this can influence reproducibility.

PMN-MDSCs and Mo-MDSCs were defined according to Bronte et al. (4) using the following gating strategy: In a dot plot of CD3 vs. CD11b, CD11b^+^ cells were gated and CD11b^+^ were then plotted in a Ly6C vs. Ly6G dot plot. PMN-MDSCs were gated as CD11b^+^Ly6CloLy6G^+^ and Mo-MDSC as CD11b^+^Ly6ChiLy6G^−^.

### Statistical Analysis

Human dataset endpoints were prespecified, while the analysis of mice was performed as *post-hoc* analysis and the results have to be considered as exploratory. For human analysis, we provide the percentage and geometric mean fluorescence intensity (MFI) data, while for mouse experiments the percentages and absolute numbers of splenocytes were available.

All datasets were tested for adherence to the Gaussian distribution with the Shapiro–Wilk test. Multiple comparisons of Gaussian-distributed data were performed using analysis of variance and Sidak's multiple comparisons test as a *post-hoc* test. The Kruskal–Wallis test with Dunn's multiple comparison test as a *post-hoc* test were used as appropriate. *Post-hoc* tests were only performed when the initial testing revealed significant differences between groups. Spearman's or Pearson's analyses were performed dependent on the adherence to the Gaussian distribution. GraphPad PRISM 8.1 (GraphPad Software Inc., San Diego, CA, USA) was used for all analyses. A *p* < 0.05 was regarded as significant.

## Results

### Human Mo-MDSCs Increase Post-stroke

**Figure 1:**
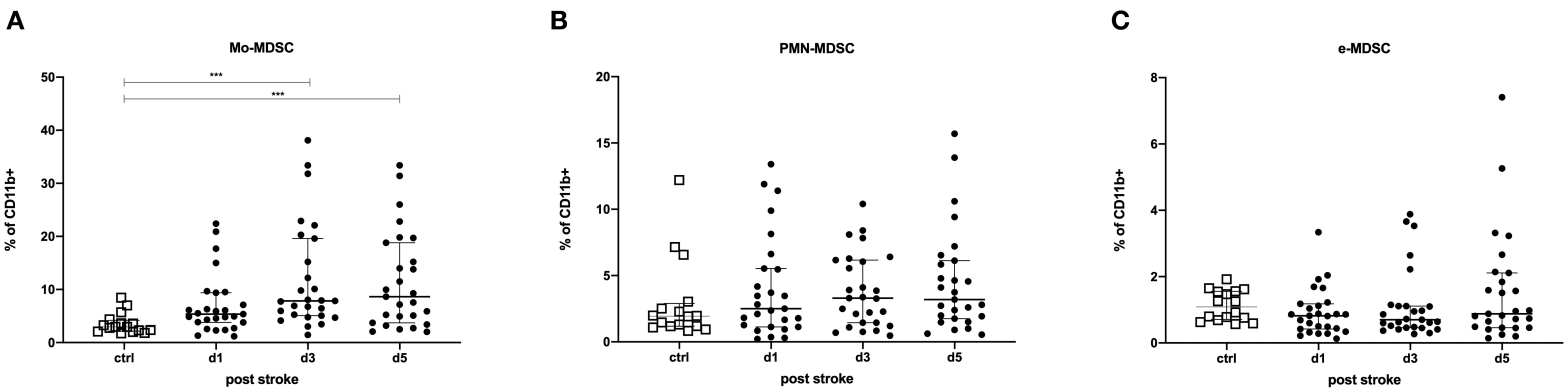
Myeloid-derived suppressor cells (MDSCs) were measured in stroke patients in comparison to healthy controls. Monocytic MDSCs (Mo-MDSC; CD11b^+^/CD14^+^/HLA-DR^dim/−^) **(A)**; granulocytic or polymorphonuclear MDSCs (PMN-MDSCs; CD11b^+^/CD15^+^/CD14^−^) **(B)**; and early MDSCs (e-MDSCs; Lin^−^/HLA-DR^−^/CD33^+^/CD11b^+^) **(C)** were measured in 27 stroke patients (*black dots*) in comparison to 16 healthy age-matched controls (*white dots*) on days 1, 3, and 5 after stroke. Cells were stained extracellularly and analyzed by flow cytometry (LSR II, BD). Median and interquartile range are provided. ***p < 0.001.

MDSCs were classified into three different subpopulations based on their surface marker expression (Supplementary Figure 1): Mo-MDSCs, PMN-MDSCs, and e-MDSCs. Mo-MDSCs (as a percentage of CD11b^+^-expressing cells) were significantly upregulated on days 3 (*p* = 0.0001) and 5 post-stroke (*p* = 0.0005) in comparison to healthy controls. A similar trend was observed for PMN-MDSCs, while e-MDSCs remained unchanged (Figure 1). The same trends were observed for the intracellular staining of the MDSC subsets (Supplementary Figure 4).

As shown in Supplementary Figures 5, 6 below, we did not observe any difference for diabetes and arterial hypertonus for Mo-MDSCs, PMN-MDSCs, and e-MDSCs.

### The Functional State of MDSC Was Not Altered Post-stroke

**Figure 2:**
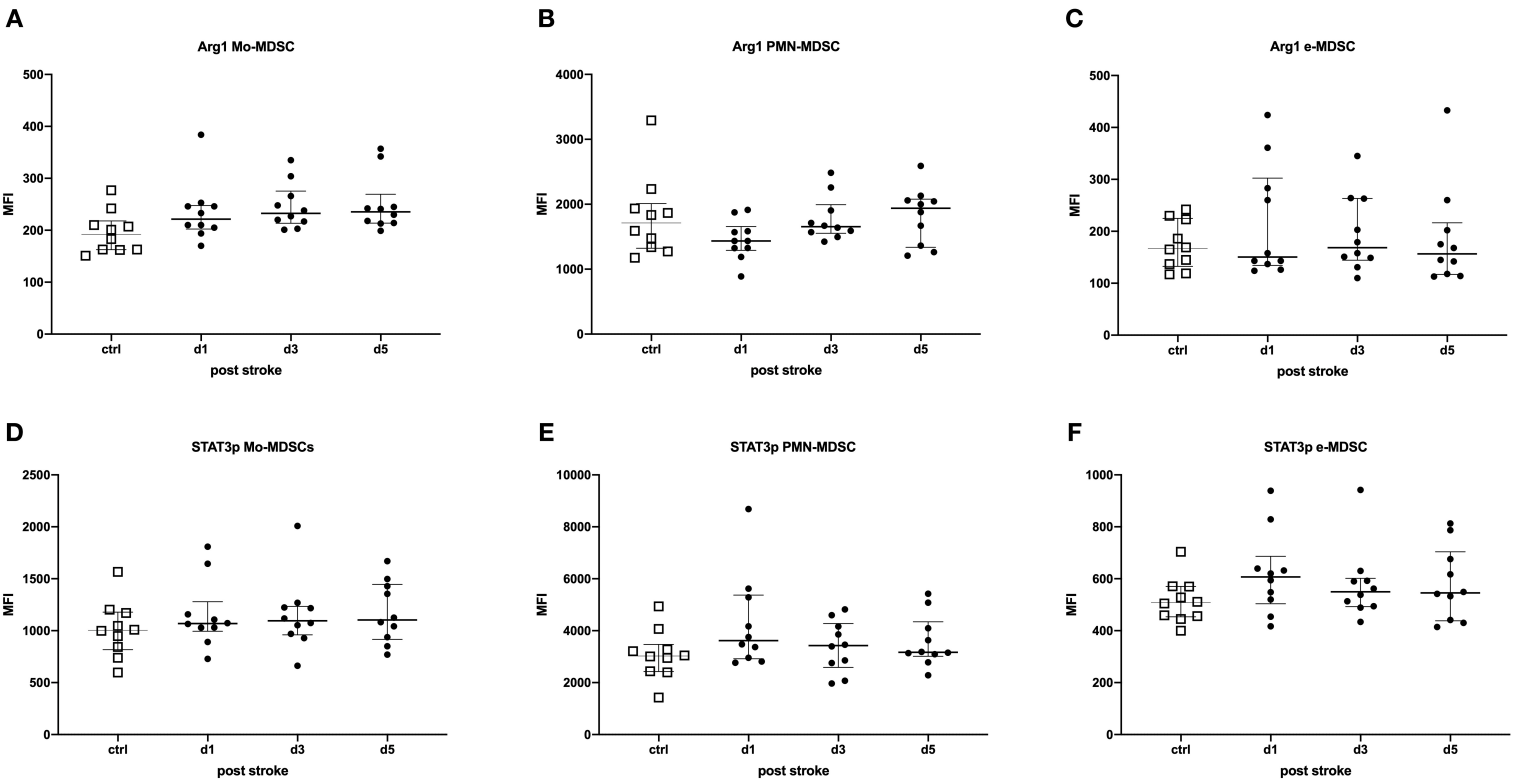
STAT3p and Arg1 were determined in myeloid-derived suppressor cell (MDSC) subpopulations of stroke patients in comparison to healthy controls. Monocytic MDSCs (Mo-MDSCs; CD11b^+^/CD14^+^/HLA-DR^dim/−^) **(A,D)**; granulocytic or polymorphonuclear MDSCs (PMN-MDSCs; CD11b^+^/CD15^+^/CD14^−^) **(B,E)**; and early MDSCs (e-MDSC; Lin^−^/HLA-DR^−^/CD33^+^/CD11b^+^) **(C,F)** were measured in 10 stroke patients (*black dots*) in comparison to 10 healthy age-matched controls (*white dots*) on days 1, 3, and 5 after stroke. Arg1 **(A–C)** and STAT3p **(D–F)** were stained intracellularly and analyzed by flow cytometry (LSR II, BD). Data are provided as geometric mean fluorescence intensity (MFI). Median and interquartile range are provided.

Intracellular STAT3p and Arg1 expressions in MDSCs were used to determine the activation status of MDSCs. The percentages of STAT3p- and Arg1-expressing MDSC subpopulations were determined and the amount of the transcription factor or the enzyme per cell was quantified by the geometric MFI. In stroke patients, these were not altered in any of the MDSCs subpopulations, suggesting an unimpaired immunosuppressive capacity of MDSCs in stroke patients (Figure 2).

Neither stroke size nor NIHSS score at discharge correlated with the percentage of MDSC subsets and the intracellular transcription factors determined. The percentage of e-MDSCs was negatively correlated with age on day 3 (*r* = −0.4096, *p* = 0.0339) and day 5 (*r* = −0.4115, *p* = 0.0329) post-stroke. The percentage of Arg1^+^ Mo-MDSCs was inversely correlated to age on day 5 (*r* = −0.7228, *p* = 0.0182) after stroke (data not shown). All other MDSC subsets as well as their transcription factors were not significantly regulated.

### Splenic Total Numbers of Mo-MDSCs and Percentage of PMN-MDSCs Increased Post-experimental Stroke

In a murine stroke model, there was no difference in the total splenocyte numbers in comparison to sham-treated animals (data not shown). Since electrocoagulation induces a relatively mild stroke, T cells, CD4^+^ T helper cells, CD8^+^ cytotoxic cells, T regulatory cells, and B cells were not altered in comparison to sham-treated samples (Supplementary Figures 7A–E). Nevertheless, CD4^+^ cells as well as B cells tended to be lower after experimental stroke (Supplementary Figures 7B,E). MHC-II on CD11b^+^ splenocytes was not altered after stroke induction (Supplementary Figure 7F).

Murine MDSCs were grouped into only two different subpopulations according to their surface markers: Mo-MDSCs (CD11b^+^Ly6G^−^Ly6C^high^) and PMN-MDSCs (CD11b^+^Ly6G^+^Ly6C^low^).

**Figure 3:**
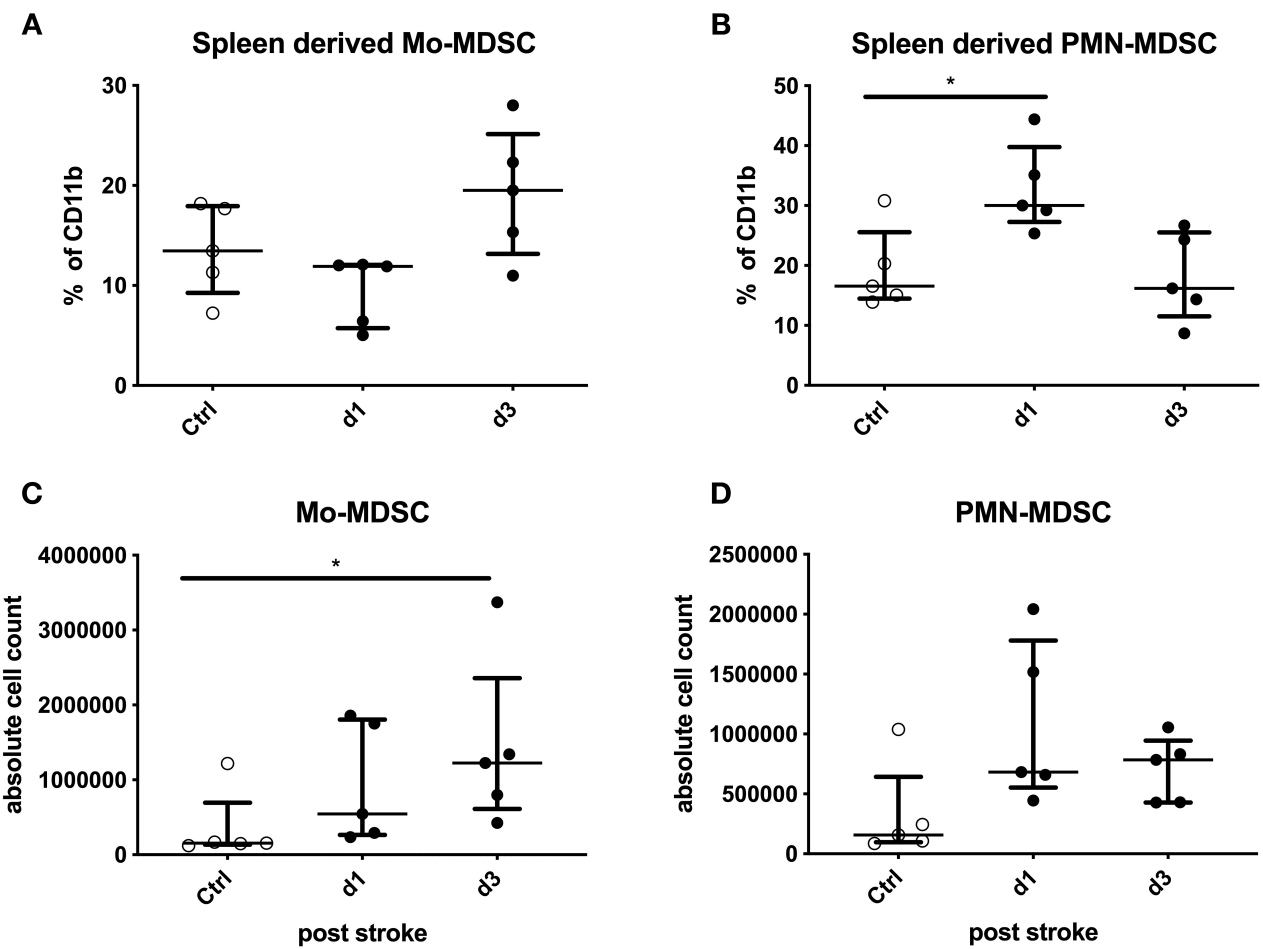
Mouse data: stroke mice are compared to sham-operated mice regarding Monocytic myeloid-derived suppressor cells (Mo-MDSCs) and granulocytic or polymorphonuclear MDSCs (PMN-MDSCs) in percentage and absolute numbers. The MDSCs of splenocytes in stroke mice (*black dots*) were analyzed in comparison to sham-operated mice (*white dots*) on days 1 and 3 after stroke induction. The percentage of IL11b^+^-expressing cells **(A,B)** and absolute numbers **(C,D)** of Mo-MDSCs **(A,C)** and PMN-MDSCs **(B,D)** were analyzed. Median and interquartile range are provided. *p < 0.05.

The percentage of Mo-MDSCs among CD11b^+^-expressing cells was not altered significantly. However, the total numbers of Mo-MDSCs from animals were significantly higher compared to the control animals on day 3 after stroke induction (*p* = 0.0324; Figures 3A,C). The percentage of PMN-MDSCs of CD11b^+^-expressing cells was increased on day 1 after stroke (*p* = 0.0238). In addition, their absolute numbers tended to be increased (*p* = 0.0568; Figures 3B,D).

Stroke lesion size neither correlated with the total numbers and percentage of PMN-MDSCs nor with Mo-MDSCs in splenocytes (data not shown).

### Monocytic Downregulation of HLA-DR and Upregulation of CD34 in Human Stroke Patients

The percentage and absolute counts of monocytes were not altered in stroke patients in comparison to the controls (data not shown).

CD34^+^Lin^−^HLA-DR^−^CD33^+^CD11b^+^CD14^+^, regulatory monocytes as described by D'Aveni et al. (18) were not detectable in the study population. We analyzed monocytes according to their expression of activation markers.

**Figure 4:**
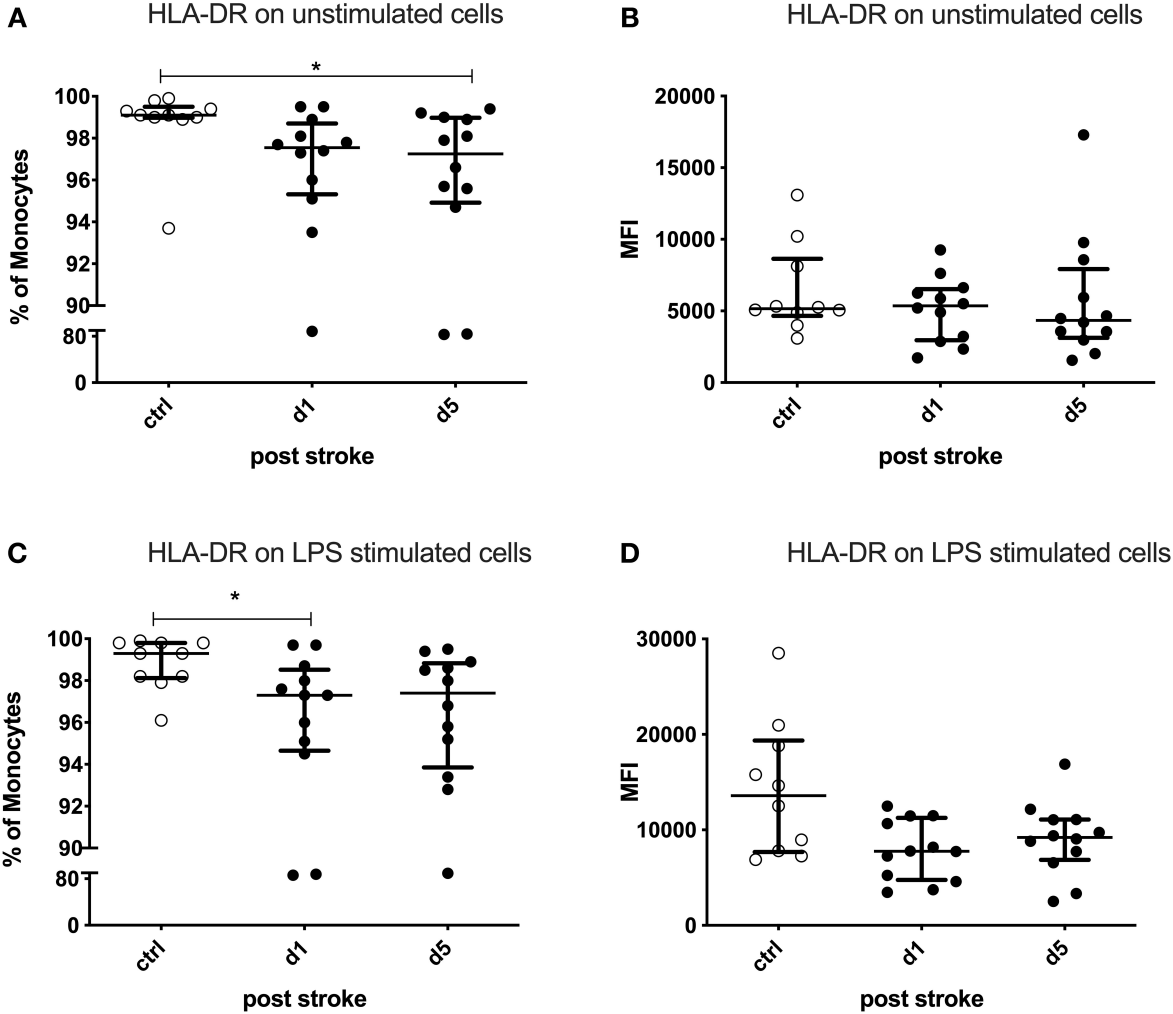
Human leukocyte antigen—DR isotype (HLA-DR) on unstimulated and lipopolysaccharide (LPS)-stimulated monocytes. Peripheral blood mononuclear cells (PBMCs) were left unstimulated **(A,B)** or treated with 10 ng/ml LPS for 20 h **(C,D)**. HLA-DR was measured as the percentage of expressing cells and the amount of HLA-DR expressed per cell—measured by the geometric mean fluorescence intensity (MFI)—in unstimulated and stimulated conditions. Measurements were performed in 12 stroke patients in comparison to 10 healthy age-matched controls. Median and interquartile range are provided. *p < 0.05.

The percentage of HLA-DR-expressing monocytes was significantly reduced on day 5 after stroke in the unstimulated condition (*p* = 0.0254). LPS-stimulated monocytes showed a defective HLA-DR upregulation already on day 1 (*p* = 0.0237; Figures 4A,C). The expression of HLA-DR on monocytes tended to be lower on day 5 in the stimulated condition (*p* = 0.0599), while the unstimulated monocytes did not show this difference (*p* = 0.5415) after stroke (Figures 4B,D).

**Figure 5:**
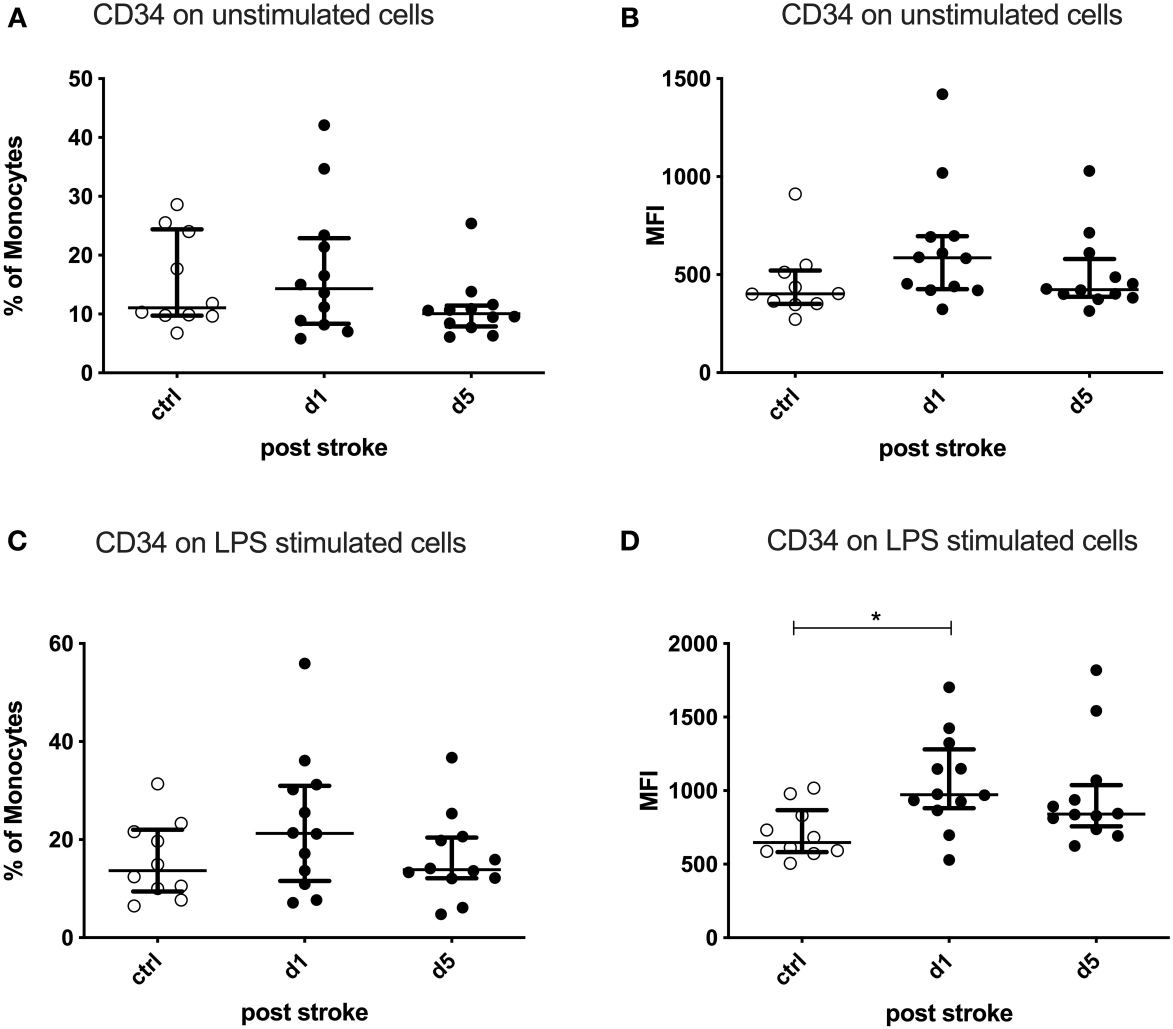
CD34 on unstimulated and lipopolysaccharide (LPS)-stimulated monocytes. Peripheral blood mononuclear cells (PBMCs) were left unstimulated **(A,B)** or treated with 10 ng/ml LPS for 20 h **(C,D)**. CD34 was measured as the percentage of expressing cells and the amount of CD34 expressed per cell—measured by the geometric mean fluorescence intensity (MFI)—in unstimulated and stimulated conditions. Measurements were performed in 12 stroke patients in comparison to 10 healthy age-matched controls. Median and interquartile range are provided. *p < 0.05.

After *ex vivo* stimulation of PBMCs by LPS, the CD34 expression per cell (MFI) was significantly upregulated on monocytes on day 1 after stroke compared to healthy controls (*p* = 0.0172). The percentage of CD34-expressing monocytes was not altered (Figure 5).

Neither the percentage of PD-L1- or PD-L2-expressing cells nor the amount of these ligands per cell was altered after stroke compared to healthy controls. The same applied to CD86 (data not shown). PD-L1 and CD86 were inversely correlated with age on day 5 after stroke in the unstimulated cells (PD-L1: *r* = −0.6344, *p* = 0.0298; CD86: *r* = −0.6658, *p* = 0.0181). All other monocyte subsets as well as their activation markers neither correlated with age nor with NIHSS (data not shown).

### IL-10^+^ and IL-10^−^ Subsets of Human Stroke Patients Differ in HLA-DR and PD-L1 Expressions

Neither the percentage of IL-10-producing monocytes nor the amount of IL-10 expression per cell was altered after stroke of human patients in the stimulated and unstimulated conditions (data not shown).

It has been reported that IL-10-producing monocytes have immune suppressive capacities (17). Therefore, we compared the expressions of HLA-DR, PD-L1, PD-L2, and CD86 in IL-10^+^ and IL-10^−^ monocytes. IL-10^−^ and IL-10^+^ monocytes were analyzed in LPS-stimulated samples.

**Figure 6:**
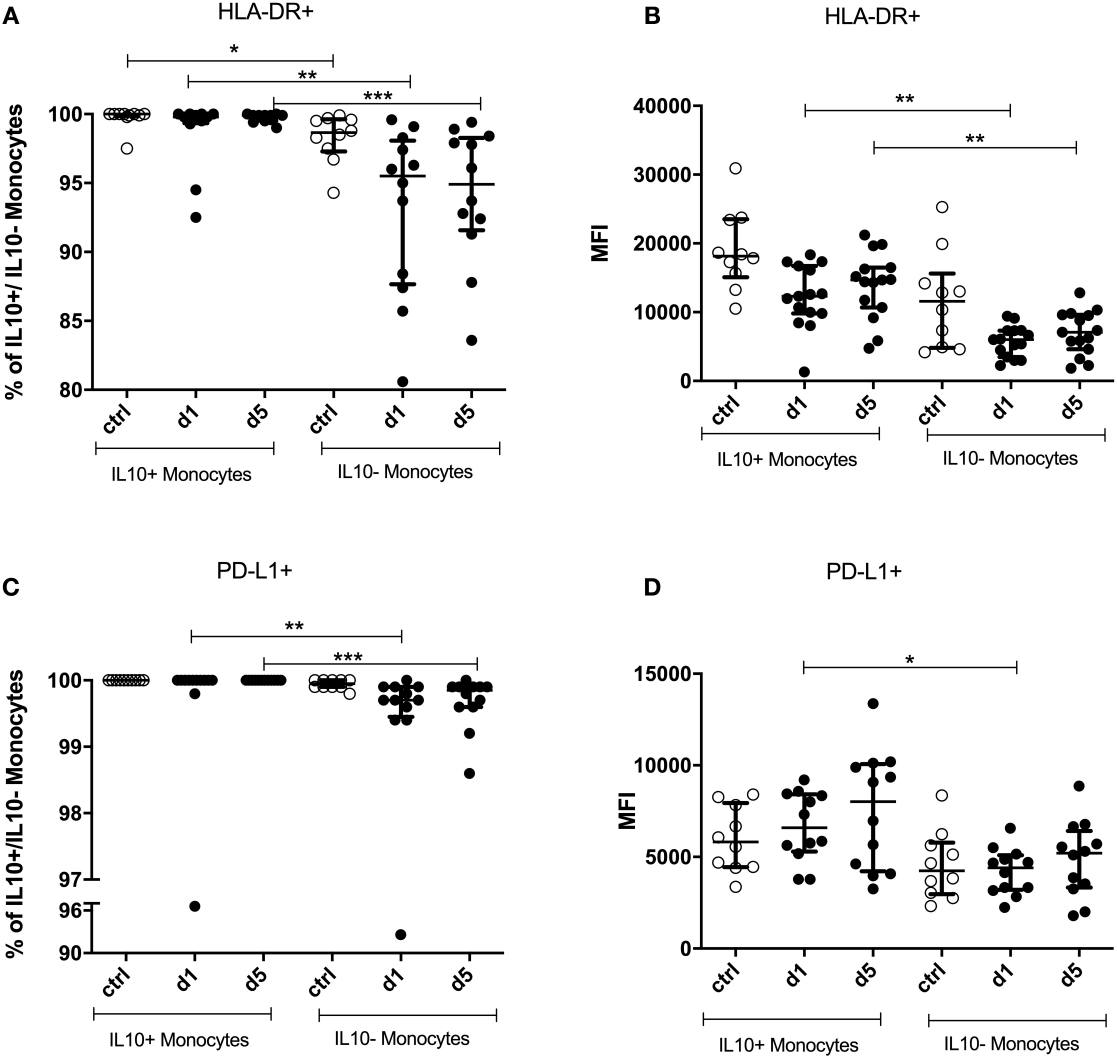
Percentage and mean fluorescence intensity (MFI) of HLA-DR^+^ and PD-L1^+^ on IL-10^+^ and IL-10^−^ monocytes. IL-10^+^ and IL-10^−^ monocytes were measured on days 1 and 5 after stroke in 12 stroke patients and 10 healthy age-matched controls. Human leukocyte antigen—DR isotype (HLA-DR) percentage **(A)** and the amount per cell **(B)**—measured by the geometric mean fluorescence intensity (MFI)—were analyzed. PD-L1 **(C,D)** was analyzed accordingly. Median and interquartile range are provided. *p < 0.05; **p < 0.01; ***p < 0.001.

Virtually all IL-10^+^ monocytes expressed HLA-DR. This proportion was significantly lower in IL-10^−^ monocytes, the difference being more pronounced in patients than in controls (Figure 6A).

Moreover, IL-10^+^ monocytes showed a significantly higher expression of HLA-DR on days 1 and 5 after stroke than did IL-10^−^ monocytes (*p* < 0.0001). Control patient samples did not differ in their expression level from patients (Figure 6B).

The percentage of PD-L1^+^ cells was higher in IL-10^+^ monocytes than in IL-10^−^ monocytes (*p* < 0.0001) only in stroke patients on days 1 and 5 (Figure 6C). The same was observed in terms of the level of PD-L1 expression, which was higher on IL-10^+^ than on IL-10^−^ monocytes on day 1 after stroke (*p* = 0.0063; Figure 6D). PD-L2 and CD86 expressions did not differ significantly between IL-10^+^ and IL-10^−^ monocytes (data not shown).

## Discussion

Since the regulation of myeloid cells during stroke-induced immune alterations is unknown, we conducted the present study to analyze the effects of stroke on MDSCs and IL-10^+^ monocytes, which are known to exert immunosuppressive properties. We observed a significant relative increase of Mo-MDSCs in patients on days 3 and 5 post-stroke in comparison to healthy controls, without any alterations in other MDSC subsets. Additionally, the experimental stroke model showed an absolute increase of Mo-MDSCs and a relative increase of PMN-MDSCs in comparison to controls. These data are in line with Liesz et al. (9) who analyzed MDSCs in post-experimental stroke and in human stroke patients 24 h after ischemia. Our results expand these findings until day 5 post-stroke and complement the analysis by STAT3p and Arg1 measurements as surrogates for suppressive function. STAT3p and Arg1 expressions were unchanged early after stroke, suggesting that the MDSCs are at least theoretically able to suppress immune responses as both the phosphorylated transcription factor STAT3 as well as the enzyme Arg1 are essential for anti-inflammatory MDSC function. We are aware that the lack of a functional suppression assay is a limitation of the current study. In mice, Liesz et al. (9) reported an increase of e-MDSC numbers in the spleen with intact suppressive function and an enhanced Arg1 expression in splenic monocytes. Our study validated the expansion of Mo-MDSCs in human ischemic stroke, while e-MDSCs were not altered and PMN-MDSCs were only increased in experimental stroke in middle-aged mice (23).

Only recently, increased levels of PMN-MDSCs and e-MDSCs were reported for patients with diabetes mellitus and arterial hypertension (23). In contrast, in our cohort, an increase of PMN-MDSCs and e-MDSCs could not be confirmed in the corresponding patient subsets. Only some patients with hypertension displayed a higher level of Mo-MDSCs in comparison to patients without hypertension. Since the number of cases per group is in part rather small (with hypertonus = 23, without hypertonus = 4; with diabetes = 10, without diabetes = 17), future studies should address this question and the possible additional impact of stroke as an acute cardiovascular event.

Mo-MDSCs were shown to play an important role in the reduction of inflammation after intraspinal transplantation of *ex vivo*-generated MDSCs at sites of spinal cord injury in mice, which led to an improvement of the neurological outcomes (24). Although immune suppression might be beneficial to prevent secondary infarct growth after stroke, the anti-inflammatory mechanisms have been shown to enhance the risk of infections and poor stroke outcomes (25). Therefore, the role of MDSCs in the post-acute stroke phase might be double-edged. Data regarding the effects of MDSCs within the brain are limited. Kawano et al. (26) reported that the counts of PMN-MDSCs were increased in the ischemic hemisphere and bone marrow at 72 h, as well as in the spleen 24 h after transient middle cerebral artery occlusion in mice. Our murine data obtained in 14-month-old mice show an increased percentage of PMN-MDSC and an enhanced Mo-MDSC count in the spleen after experimental stroke. Whether Mo-MDSCs might also have an inhibitory potential on T cell activation in ischemic brain should be addressed in future studies.

Stroke induces a higher number of circulating CD34^+^ cells during the first 6 days after ischemic insult (5). Nevertheless, the number of clonogeneic CD34 cells released by stroke is much lower than that observed after pharmacological mobilizations by G-CSF of cyclophosphamide. D'Aveni et al. (18) collected peripheral blood hematopoietic stem cells (PBSCs) mobilized by filgrastim (10 mg kg^−1^ day^−1^, 5 consecutive days) from healthy donors for allogeneic hematopoietic stem cell transplantation (allo-HSCT) when they reported about CD34^+^-suppressive cells. This may represent a different cell population, which might explain why we were not able to detect this subset in the peripheral blood of healthy donors and stroke patients.

HLA-DR is important to present peptide antigens for the purpose of eliciting or suppressing T (helper) cell responses. The downregulation of HLA-DR in monocytes has previously been identified as a prototypical biomarker for stroke-induced immune suppression (SIIS) and stroke outcomes (15, 27–29). Our new data show that this reduction is restricted to IL-10^−^ monocytes, while IL-10-expressing monocytes remain fully HLA-DR competent after stroke. Macrophages derived from IL-10-secreting monocytes are capable of augmenting TH2 immune responses and inhibiting T cell proliferation (30–32). The induction of SIIS is known to be multifactorial, and IL-10^+^-producing monocytes could be among the inducers of immune suppression within the innate immune cell compartment.

PD-L1, expressed on hematopoietic and parenchymal cells, binds to the programmed death-1 (PD-1) receptor to inhibit T cell receptor-mediated proliferation and to induce T cell anergy. Engagement of the PD-1 pathway is essential in suppressing autoimmunity, as originally demonstrated in mice lacking PD-1 expression that developed spontaneous autoimmune disease (33). Furthermore, PD-L2, through engaging with PD-1, negatively regulates T cells in immune responses and plays an essential role in immune tolerance (34). We found that PD-L1 was expressed at higher levels in IL-10^+^ than in IL-10^−^ monocytes. PD-L1 is a checkpoint regulator of the adaptive immune response as it can induce T cell anergy, cytostasis, and apoptosis. Only recently, Ly6C^low^ CX3CR1^high^, patrolling “non-classical” murine monocytes expressing PD-L1, were shown to induce apoptosis in T cells, especially in tertiary lymphoid organs (35). Therefore, PDL-1 could provide additive effects in IL-10^+^ monocytes in SIIS.

Sepsis stimulates emergency myelopoiesis, which induces the expansion of MDSCs (36) Brudecki et al. (37) demonstrated that during the early phase, MDSCs secreted nitric oxide (NO) and pro-inflammatory cytokines, whereas in the later chronic phase, MDSCs expressed Arg1 and IL-10 proteins in a mouse polymicrobial sepsis model. Consequently, these MDSCs induce a profound immunosuppression, which might be comparable to that present after stroke.

However, MDSCs underlie a complex regulation after stroke in which immunosenescence of the typical aged stroke patient and stroke effects might have opposing effects: the percentages of MDSCs were significantly increased in the blood of old people (80–100 years) in comparison to a young control group (20–30 years) (38). Furthermore, CD11b^+^CD15^+^ PMN-MDSCs were increased in the blood of seniors (61–76 years), and especially in frail elderly people (67–99 years) (39). In contrast, our data partly show an inverse correlation with age (e-MDSCs on days 3 and 5 after stroke and Arg1 on Mo-MDSCs on day 5).

### Limitations

One limitation of this study is that the limited amount of blood that could be obtained from stroke patients did not allow us to test the suppressive properties of our bona fide MDSC subpopulations with an *in vitro* T cell suppression assay. According to the algorithm by Bronte et al. (4) the phenotyping of MDSCs is followed by showing the suppressive properties in functional assays, which is then followed by the analysis of functionally important intracellular targets. Therefore, we analyzed STAT3p (40) and Arg1 as two functionally important intracellular targets as surrogates for suppressive capacity, as was published before by Bruger et al. (41). This attempt still could not verify functional suppression. In addition, other effector mechanisms of MDSCs like inducible NO synthase (iNOS), ROS, or 2,3-dioxygenase (IDO) could be included to broaden the information.

Due to the small number of animals (*n* = 5) within the *post-hoc* analysis, the results are of an exploratory nature. Future experiments should verify the trends within larger cohorts.

## Conclusion

In order to investigate the potential immunosuppressive role of MDSCs after stroke, the transcription factor STAT3p as well as Arg1 were investigated, which are both involved in MDSC-induced T cell suppression. The results support the notion of an immunosuppressive involvement of Mo-MDSCs: their percentages were increased post-stroke and STAT3p expression was confirmed.

Interestingly, HLA-DR was downregulated in IL-10^−^ monocytes, but not in IL-10^+^ monocytes. The latter cell subpopulation could present antigen to specific CD4^+^ T cells and dampen their inflammatory function *via* IL-10, thereby contributing to immune suppression after stroke.

## Data Availability Statement

The raw data supporting the conclusions of this article will be made available by the authors, without undue reservation.

## Ethics Statement

The study protocol was approved by the ethics committee of the Medical Faculty, University of Greifswald (BB 041/17a,b,c). The patients/participants provided their written informed consent to participate in this study. Patients gave consent directly or through a surrogate. The animal study was reviewed and approved by Landesamt für Landwirtschaft, Lebensmittelsicherheit und Fischerei (LALLF), Mecklenburg-Vorpommern (Nr. 7221.3-1-056/15).

## Author Contributions

AV, JS, JR, and AD made substantial contributions to the conception and design, data analysis, interpretation of the data, and were involved in drafting the manuscript and revising it critically for important intellectual content. LA, JS, BS, and BB made substantial contributions to the acquisition of patients or data, data analysis, and interpretation of the data. SG evaluated stroke lesion sizes. All authors have read and approved the final manuscript.

## Conflict of Interest

The authors declare that the research was conducted in the absence of any commercial or financial relationships that could be construed as a potential conflict of interest.

## Supplementary Material

Supplementary Figure 1 Schematic representation of the origin of MDSCs. In certain conditions, cytokines, and stimulating factors promote the expansion of MDSCs from immature myeloid precursors. MDSCs themselves are still rather immature cells that acquire immunosuppressive functions. Based on the expression of extracellular markers, three major subsets of human MDSCs [compare Bronte et al. (4)] can be identified.

Supplementary Figure 2 Gating strategy for human MDSC subsets (Mo-MDSCs, PMN-MDSCs, and e-MDSCs) and intracellular staining of Arginase-1 and STAT3p expression. **(A)** Following single cell gating in both FSC-H/FSC-W and SSC-H/SSC-W, human MDSC subsets are defined within the CD11b+ gate. Mo-MDSCs were defined as CD11b+/CD14+/HLA-DRdim/–, PMN-MDSCs as CD11b+/CD15+/CD14– and e-MDSCs as Lin-/HLA-DR-/CD33+/CD11b+ **(B)** Histograms of intracellular expression of Arginase-1 and STAT3p in human MDSC subsets. For the detection of intracellular Arginase-1 and STAT3p expression, FMO controls (light gray) were used for each MDSC subset.

Supplementary Figure 3 Gating strategy for human monocytes and expression of intracellular and extracellular markers in stimulated and unstimulated conditions. **(A)** Following single cell gating (FSC-H/FSC-A; SSC-H/SSC-A) and dead cell exclusion, human monocytes were defined based on the expression of CD14 and CD11b as well as their relative representation in SSC-A. **(B)** Pseudocolor dot plot (B/W) of IL-10 expression in unstimulated and stimulated conditions. Gating for IL-10^+^ monocytes was defined within the unstimulated condition and then applied to the stimulated condition to discriminate IL-10^+^ monocytes within. **(C)** Histograms of extracellular marker expression (dark gray) of human monocytes. Corresponding FMO controls (light gray) were used for detection. Marker expression in IL-10^+^ and IL-10—monocyte subsets was determined accordingly.

Supplementary Figure 4 MDSC were measured in stroke patients in comparison to healthy controls (intracellular staining). Mo-MDSC (CD11b+/CD14+/HLA-DRdim/–) **(A)**; PNM-MDSC (CD11b+/CD15+/CD14–) **(B)** and e-MDSC (Lin-/HLA-DR-/CD33+/CD11b+) **(C)** were measured in 10 stroke patients (black dots) in comparison to 10 healthy age-matched controls (white dots) on day 1, 3, and 5 after stroke. Cells were stained intracellularly and analyzed by flow cytometry LSR II (BD). Median and interquartile range is provided. ^*^*p* < 0.05; ^**^*p* < 0.01; ^***^*p* < 0.001.

Supplementary Figure 5 MDSC subsets regarding the occurrence of arterial hypertonus. Mo-MDSC (CD11b+/CD14+/HLA-DRdim/–) **(A)**; PNM-MDSC (CD11b+/CD15+/CD14–) **(B)** and e-MDSC (Lin-/HLA-DR-/CD33+/CD11b+) **(C)** on day 1, 3, and 5 after stroke regarding the occurrence of arterial hypertonus—with hypertonus (black dots), without hypertonus (white squares). Values are given as percentages. Median and interquartile range is provided.

Supplementary Figure 6 MDSC subsets regarding the occurrence of diabetes mellitus. Mo-MDSC (CD11b+/CD14+/HLA-DRdim/–) **(A)**; PNM-MDSC (CD11b+/CD15+/CD14–) **(B)** and e-MDSC (Lin-/HLA-DR-/CD33+/CD11b+) (C) on day 1, 3, and 5 after stroke regarding the occurrence of diabetes mellitus—with diabetes mellitus (black dots), without diabetes mellitus (white squares). Values are given as percentages. Median and interquartile range is provided.

Supplementary Figure 7 T-cell subpopulation in experimental stroke. T-cell **(A)** and their subpopulations—CD4+**(B)**, CD8+ **(C)**, and T-regulatory cells **(D)** as well as B-cells **(E)** were stained in mouse splenocytes after experimental stroke in comparison to sham-treated animals. Additionally, MHC-II on CD11b+ splenocytes were measured **(F)**. Values are given as percentages. Median and interquartile range is provided. ^*^*p* < 0.05.

Supplementary Table 1 Patient characteristic of MDSC extracellular staining. Patients' blood was stained with anti-CD11b BV510, anti-CD33 BV421, anti-CD14 PE/Cy7, anti-CD15 AF700, anti-HLA-DR FITC, and anti-Lineage Cocktail APC (anti- CD3/CD14/CD16/CD19/CD20/CD56) extracellularly. Patients characteristics are given in the following table.

Supplementary Table 2 Patient characteristic of MDSC intracellular staining. Patients' blood was stained with anti-Arginase-1 PE and anti-STAT3 phospho PerCP/Cy5.5 intracellularly. Patients characteristics are given in the following table.

Supplementary Table 3 Patient characteristic of regulatory monocyte staining. Patients' blood was stained with anti-CD11b BV510, anti-CD34 BV421, anti-CD14 APC/Cy7, anti-CD86 BV650, anti-HLA-DR FITC, anti-CD33 PerCP/Cy5.5, anti-PD-L1 PE/Dazzle594, anti-PD-L2 APC, and anti-IL-10 PE for description of regulatory monocytes. Patients characteristics are given in the following table.
