# Supplementary figures and images for: Stroke-Induced Modulation of Myeloid-Derived Suppressor Cells (MDSCs) and IL-10-Producing Regulatory Monocytes
Source: Front Neurol. 2020 Nov 25;11:577971. doi: 10.3389/fneur.2020.577971 (PMC7732608; doi:10.3389/fneur.2020.577971)

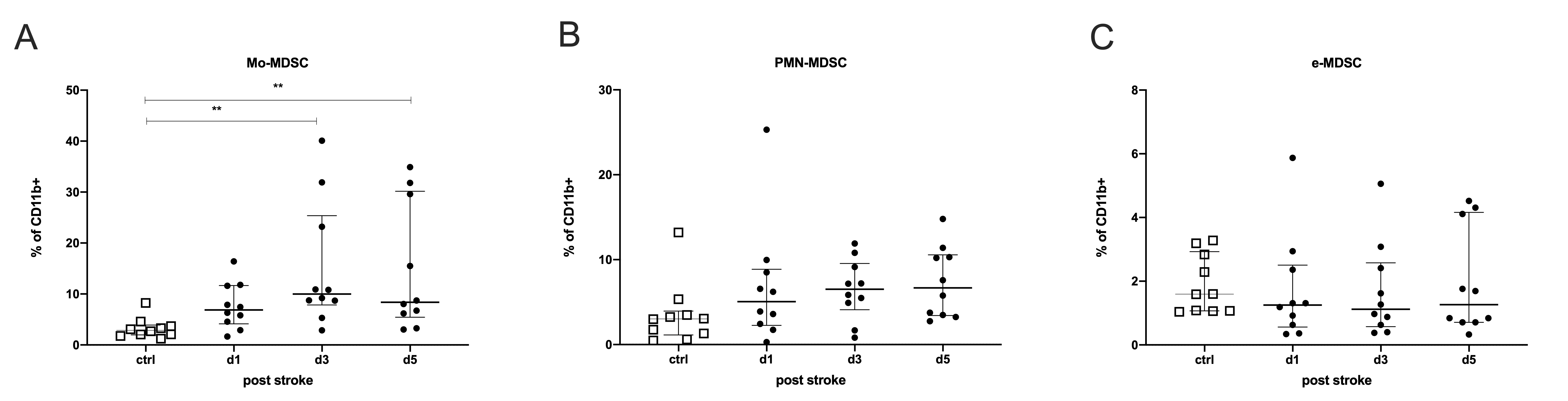

Supplement: Supplementary Figure 4 — MDSC were measured in stroke patients in comparison to healthy controls (intracellular staining). Mo-MDSC (CD11b+/CD14+/HLA-DRdim/–) (A); PNM-MDSC (CD11b+/CD15+/CD14–) (B) and e-MDSC (Lin-/HLA-DR-/CD33+/CD11b+) (C) were measured in 10 stroke patients (black dots) in comparison to 10 healthy age-matched controls (white dots) on day 1, 3, and 5 after stroke. Cells were stained intracellularly and analyzed by flow cytometry LSR II (BD). Median and interquartile range is provided. *p < 0.05; **p < 0.01; ***p < 0.001. [file Image_4.jpg]

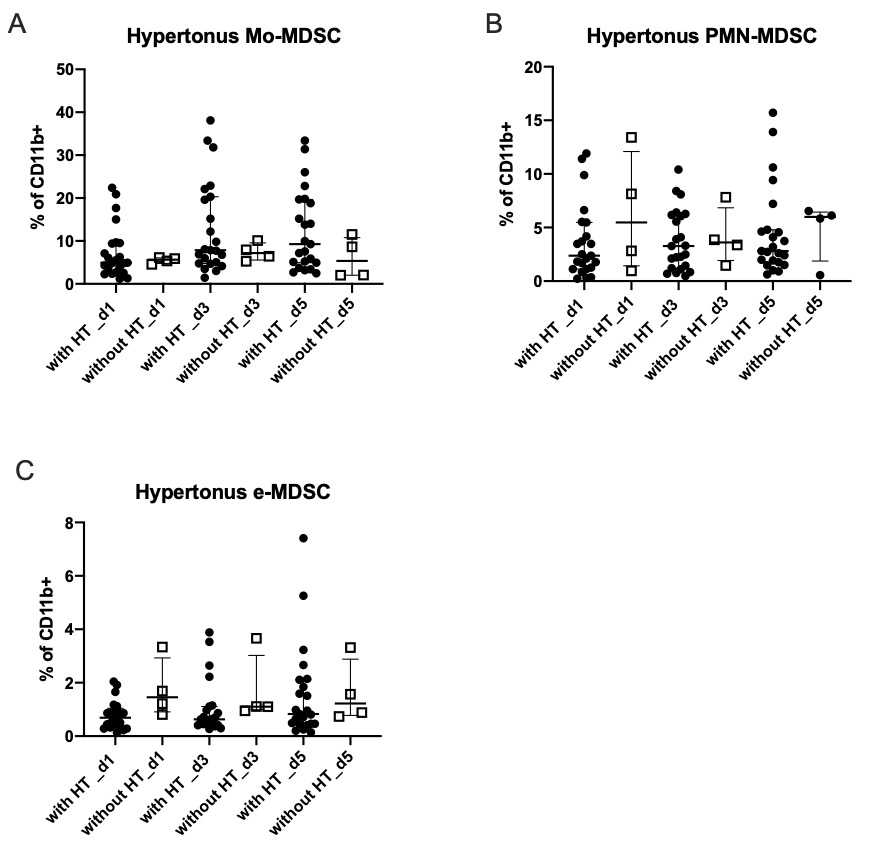

Supplement: Supplementary Figure 5 — MDSC subsets regarding the occurrence of arterial hypertonus. Mo-MDSC (CD11b+/CD14+/HLA-DRdim/–) (A); PNM-MDSC (CD11b+/CD15+/CD14–) (B) and e-MDSC (Lin-/HLA-DR-/CD33+/CD11b+) (C) on day 1, 3, and 5 after stroke regarding the occurrence of arterial hypertonus—with hypertonus (black dots), without hypertonus (white squares). Values are given as percentages. Median and interquartile range is provided. [file Image_5.jpg]

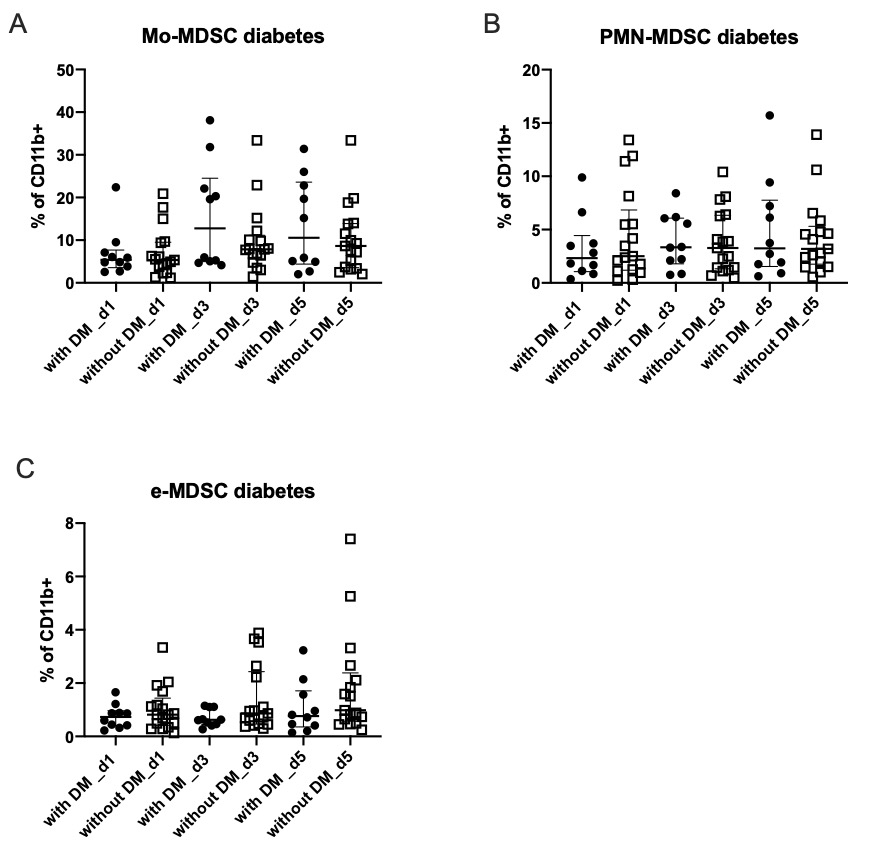

Supplement: Supplementary Figure 6 — MDSC subsets regarding the occurrence of diabetes mellitus. Mo-MDSC (CD11b+/CD14+/HLA-DRdim/–) (A); PNM-MDSC (CD11b+/CD15+/CD14–) (B) and e-MDSC (Lin-/HLA-DR-/CD33+/CD11b+) (C) on day 1, 3, and 5 after stroke regarding the occurrence of diabetes mellitus—with diabetes mellitus (black dots), without diabetes mellitus (white squares). Values are given as percentages. Median and interquartile range is provided. [file Image_6.jpg]

A

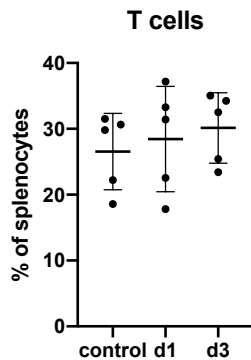

B

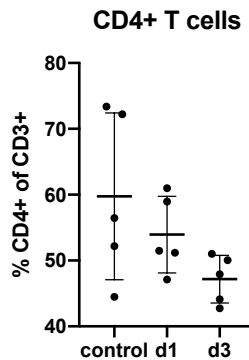

C

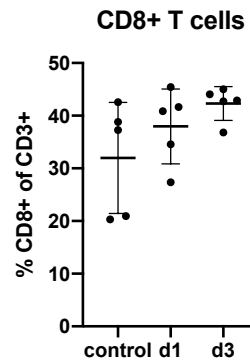

D

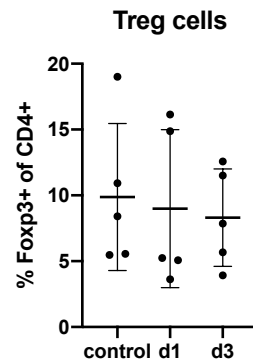

E

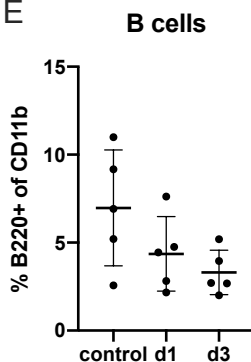

F

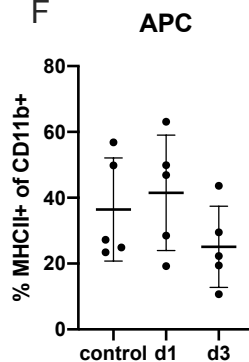

Supplement: Supplementary Figure 7 — T-cell subpopulation in experimental stroke. T-cell (A) and their subpopulations—CD4+(B), CD8+ (C), and T-regulatory cells (D) as well as B-cells (E) were stained in mouse splenocytes after experimental stroke in comparison to sham-treated animals. Additionally, MHC-II on CD11b+ splenocytes were measured (F). Values are given as percentages. Median and interquartile range is provided. *p < 0.05. [file Image_7.pdf]
