# Supplementary material for: Stroke-Induced Modulation of Myeloid-Derived Suppressor Cells (MDSCs) and IL-10-Producing Regulatory Monocytes
Source: Front Neurol. 2020 Nov 25;11:577971. doi: 10.3389/fneur.2020.577971 (PMC7732608; doi:10.3389/fneur.2020.577971)
Supplement: Supplementary Table 1 — Patient characteristic of MDSC extracellular staining. Patients' blood was stained with anti-CD11b BV510, anti-CD33 BV421, anti-CD14 PE/Cy7, anti-CD15 AF700, anti-HLA-DR FITC, and anti-Lineage Cocktail APC (anti- CD3/CD14/CD16/CD19/CD20/CD56) extracellularly. Patients characteristics are given in the following table. [file Table_1.docx]

Suppl. Table 1

| Variable | Patient Group (N=27) | Control Group (N=16) |  |
| --- | --- | --- | --- |
| Age [Years, Mean ± SD] | 70,2 ± 13,4 | 67,4 ± 10,1 |  |
| Sex [as % female] | 40,7 | 37,5 |  |
| Co-morbidities |  |  |  |
| Hypertension [n (%)] | 23 (85,19) | 12 (75,00) |  |
| Diabetes mellitus [n (%)] | 10 (37,04) | 6 (37,5) |  |
| Stroke Characteristics |  |  |  |
| Etiology |  |  |  |
| Large-artery atherosclerosis [n (%)] | 3 (11,11) | NA^$^ |  |
| Cardio embolism [n (%)] | 9 (33,33) | NA^$^ |  |
| Stroke of other determined etiology [n (%)] | 5 (18,52) | NA^$^ |  |
| Stroke of undetermined etiology [n (%)] | 10 (37,04) | NA^$^ |  |
| 1. MRI Stroke Size* [ml3, Median (IQR)] | 2,88 (4,83) | NA^$^ |  |
| Initial NIHSS [Median (IQR)] | 11 (7) | NA^$^ |  |
| NIHSS at discharge [Median (IQR)] | 4 (7,5) | NA^$^ |  |
| Infarct side [n (%) left sided infarcts] | 19 (70,37) | NA^$^ |  |
| Treatment [n (%)] | 19 (70,37) | NA^$^ |  |
| Systemic Thrombolysis [n (%)]^&^ | 17 (89,47) | NA^$^ |  |
| Mechanical Thrombectomy [n (%)]^&^ | 10 (52,63) | NA^$^ |  |
| Combined Treatment [n (%)]^&^ | 8 (42,11) | NA^$^ |  |
|  |  |  |  |
| ^&^The numbers of systemic thrombolysis and mechanical thrombectomies  are the total number of patients receiving the treatments and include patients receiving  a combination of both. ^$^NA: Not applicable. * Stroke size could be determined in 7 out of the 27 patients. | | |  |
|  |  |  |  |
|  |  |  |  |
|  |  |  |  |
