# Supplementary material for: Stroke-Induced Modulation of Myeloid-Derived Suppressor Cells (MDSCs) and IL-10-Producing Regulatory Monocytes
Source: Front Neurol. 2020 Nov 25;11:577971. doi: 10.3389/fneur.2020.577971 (PMC7732608; doi:10.3389/fneur.2020.577971)
Supplement: Supplementary Table 2 — Patient characteristic of MDSC intracellular staining. Patients' blood was stained with anti-Arginase-1 PE and anti-STAT3 phospho PerCP/Cy5.5 intracellularly. Patients characteristics are given in the following table. [file Table_2.docx]

Suppl. Table 2

| Variable | Patient Group (N=10) | Control Group (N=10) |  |
| --- | --- | --- | --- |
| Age [Years, Mean ± SD] | 66,6 ± 10,7 | 69,4 ± 9,5 |  |
| Sex [as % female] | 40 | 40 |  |
| Co-morbidities |  |  |  |
| Hypertension [n (%)] | 7 (70,00) | 9 (90,00) |  |
| Diabetes mellitus [n (%)] | 4 (40,00) | 3 (30,00) |  |
| Stroke Characteristics |  |  |  |
| Etiology |  |  |  |
| Large-artery atherosclerosis [n (%)] | 0 (0) | NA^$^ |  |
| Cardio embolism [n (%)] | 3 (30,00) | NA^$^ |  |
| Stroke of other determined etiology [n (%)] | 2 (20,00) | NA^$^ |  |
| Stroke of undetermined etiology [n (%)] | 5 (50,00) | NA^$^ |  |
| 1. MRI Stroke Size* [ml3, Median (IQR)] | 4,1 (3,2) | NA^$^ |  |
| Initial NIHSS [Median (IQR)] | 10,5 (4,75) | NA^$^ |  |
| NIHSS at discharge [Median (IQR)] | 4,5 (5,75) |  |  |
| Infarct side [n (%) left sided infarcts] | 5 (50,00) | NA^$^ |  |
| Treatment [n (%)] | 9 (90,00) | NA^$^ |  |
| Systemic Thrombolysis [n (%)]^&^ | 8 (88,89) | NA^$^ |  |
| Mechanical Thrombectomy [n (%)]^&^ | 6 (66,67) | NA^$^ |  |
| Combined Treatment [n (%)]^&^ | 5 (55,56) | NA^$^ |  |
|  |  |  |  |
| ^&^The numbers of systemic thrombolysis and mechanical thrombectomies  are the total number of patients receiving the treatments and include patients receiving  a combination of both. ^$^NA: Not applicable. * Stroke size could be determined in 2 out of the 10 patients. | | |  |
|  |  |  |  |
|  |  |  |  |
|  |  |  |  |
