# Supplementary material for: Stroke-Induced Modulation of Myeloid-Derived Suppressor Cells (MDSCs) and IL-10-Producing Regulatory Monocytes
Source: Front Neurol. 2020 Nov 25;11:577971. doi: 10.3389/fneur.2020.577971 (PMC7732608; doi:10.3389/fneur.2020.577971)
Supplement: Supplementary Table 3 — Patient characteristic of regulatory monocyte staining. Patients' blood was stained with anti-CD11b BV510, anti-CD34 BV421, anti-CD14 APC/Cy7, anti-CD86 BV650, anti-HLA-DR FITC, anti-CD33 PerCP/Cy5.5, anti-PD-L1 PE/Dazzle594, anti-PD-L2 APC, and anti-IL-10 PE for description of regulatory monocytes. Patients characteristics are given in the following table. [file Table_3.docx]

Suppl. Table 3

| Variable | Patient Group (N=12) | Control Group (N=10) |  |
| --- | --- | --- | --- |
| Age [Years, Mean ± SD] | 69,9 ± 12,9 | 67,6 ± 7,7 |  |
| Sex [as % female] | 25 | 30 |  |
| Co-morbidities |  |  |  |
| Hypertension [n (%)] | 8 (66,67) | 9 (90,00) |  |
| Diabetes mellitus [n (%)] | 4 (33,33) | 5 (50,00) |  |
| Stroke Characteristics |  |  |  |
| Etiology |  |  |  |
| Large-artery atherosclerosis [n (%)] | 2 (16,67) | NA^$^ |  |
| Cardio embolism [n (%)] | 4 (33,33) | NA^$^ |  |
| Stroke of other determined etiology [n (%)] | 2 (16,67) | NA^$^ |  |
| Stroke of undetermined etiology [n (%)] | 4 (33,33) | NA^$^ |  |
| 1. MRI Stroke Size* [ml3, Median (IQR)] | 4,21 (3,1) | NA^$^ |  |
| Initial NIHSS [Median (IQR)] | 10 (3,25) | NA^$^ |  |
| NIHSS at discharge [Median (IQR)] | 3,5 (3,75) |  |  |
| Infarct side [n (%) left sided infarcts] | 7 (58,33) | NA^$^ |  |
| Treatment [n (%)] | 9 (75,00) | NA^$^ |  |
| Systemic Thrombolysis [n (%)]^&^ | 9 (100,00) | NA^$^ |  |
| Mechanical Thrombectomy [n (%)]^&^ | 5 (55,56) | NA^$^ |  |
| Combined Treatment [n (%)]^&^ | 5 (55,56) | NA^$^ |  |
|  |  |  |  |
| ^&^The numbers of systemic thrombolysis and mechanical thrombectomies  are the total number of patients receiving the treatments and include patients receiving  a combination of both. ^$^NA: Not applicable. * Stroke size could be determined in 2 out of the 12 patients. | | |  |
|  |  |  |  |
|  |  |  |  |
|  |  |  |  |
